# Cardiomyocyte proliferation and regeneration in congenital heart disease

**DOI:** 10.1002/pdi3.2501

**Published:** 2024-08-12

**Authors:** Jialiang Liang, Xingyu He, Yigang Wang

**Affiliations:** ^1^ Department of Pathology and Laboratory Medicine College of Medicine University of Cincinnati Cincinnati Ohio USA

**Keywords:** cardiomyocytes, congenital heart disease, proliferation, regenerative medicine

## Abstract

Despite advances in prenatal screening and a notable decrease in mortality rates, congenital heart disease (CHD) remains the most prevalent congenital disorder in newborns globally. Current therapeutic surgical approaches face challenges due to the significant rise in complications and disabilities. Emerging cardiac regenerative therapies offer promising adjuncts for CHD treatment. One novel avenue involves investigating methods to stimulate cardiomyocyte proliferation. However, the mechanism of altered cardiomyocyte proliferation in CHD is not fully understood, and there are few feasible approaches to stimulate cardiomyocyte cell cycling for optimal healing in CHD patients. In this review, we explore recent progress in understanding genetic and epigenetic mechanisms underlying defective cardiomyocyte proliferation in CHD from development through birth. Targeting cell cycle pathways shows promise for enhancing cardiomyocyte cytokinesis, division, and regeneration to repair heart defects. Advancements in human disease modeling techniques, clustered regularly interspaced short palindromic repeats ‐based genome and epigenome editing, and next‐generation sequencing technologies will expedite the exploration of abnormal machinery governing cardiomyocyte differentiation, proliferation, and maturation across diverse genetic backgrounds of CHD. Ongoing studies on screening drugs that regulate cell cycling are poised to translate this nascent technology of enhancing cardiomyocyte proliferation into a new therapeutic paradigm for CHD surgical interventions.

## 1 INTRODUCTION

**FIGURE 1:**
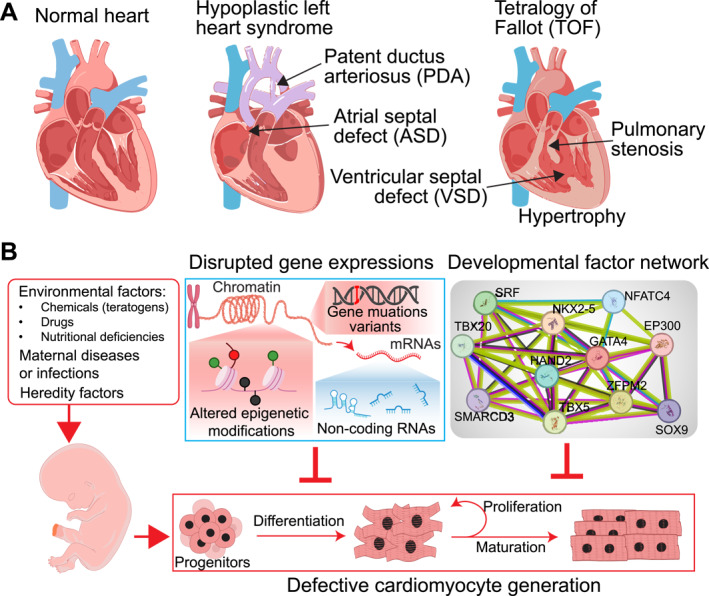
Schematic diagram of CHD malformations and underlying mechanisms. (A): In comparison to a normal heart, the abnormal structures in CHD cases are evident in the connections between the left and right chambers or vessels, morphological abnormalities, and structural defects. (B): CHD is associated with multilayered mechanisms, including chromosomal aberrations, altered epigenetic mechanisms, genetic variations or mutations, transcriptomic changes, noncoding RNA modulations, disturbed transcription factor interactions, and environmental factors. CHD, congenital heart disease.

Congenital heart disease (CHD), the most common cause of infant mortality, encompasses a varied range of structural and functional heart abnormalities that originate during fetal gestation and are present at birth.^1^ These diverse disorders impact the heart chambers, valves, or blood vessels, ranging from isolated atrial or ventricular defects to intricate malformations, such as the total absence of a cardiac chamber or the transposition of major blood vessels.^2^ Advancements in diagnosis and monitoring through advanced imaging techniques, biomarkers, and devices, coupled with enhancements in percutaneous and surgical interventions and the optimization of care organization, have facilitated the transition of the majority of patients into adulthood.^3^, ^4^ A commonly utilized classification scheme distinguishes CHD into categories of great complexity, moderate severity, and simplicity based on the recommended frequency of specialized center visits for adult CHD patients, yet there is no comparable clinical classification specifically tailored to pediatric CHD cases.^5^ The most prevalent subtypes of CHD include ventricular septal defect (VSD), atrial septal defect (ASD), patent ductus arteriosus (PDA), and tetralogy of Fallot (TOF) (Figure 1A).^6^ Interestingly, left ventricle ejection fraction has been proposed as a valuable indicator for classifying pediatric heart failure and aiding in the management of CHD.^7^

CHD arises from a complex interplay of genetic (such as DNA mutations and copy number variants), environmental, and multifactorial factors during fetal development (Figure 1B).^8^, ^9^, ^10^ Despite advancements in prenatal screening techniques and a significant decline in mortality rates, CHD persists as the most prevalent congenital disorder in newborns, with the global number of CHD patients continuing to rise.^11^ CHD treatment encompasses a multidisciplinary approach tailored to address the structural and functional abnormalities present from birth, mainly involving a combination of medical management, cardiac devices, surgical interventions, catheter‐based procedures, lifestyle modifications, and long‐term monitoring to optimize cardiac function and overall well‐being.^2^, ^12^ Despite the prolonged survival of CHD patients due to these therapeutic advancements, many patients still experience late complications such as arrhythmias, heart failure, endocarditis, and pulmonary hypertension, leading to the development of cardiac morbidities and often requiring (re)interventions/operations or eventual heart transplantation.^5^, ^12^ Thus, the continued pursuit of innovative therapies remains essential to safeguard cardiac function, diminish morbidity, and enhance overall outcomes for patients with CHD.

Notably, emerging cardiac regenerative approaches offer promising adjunct therapeutics for treating CHD through techniques such as transplantation of stem cells. Various types of stem cells, including cardiosphere‐derived cells, mesenchymal stem cells, cardiac stem/progenitor cells, and endothelial progenitor cells, are currently under evaluation in preclinical studies or clinical trials, demonstrating their safety and potential for improving cardiac function in CHD treatment.^13^, ^14^, ^15^ Crucially, human‐induced pluripotent stem cells (iPSCs), akin to embryonic stem cells, can be generated from human somatic cells through cellular reprogramming techniques, providing an abundant and unrestricted cell reservoir for cardiovascular cells for regenerative therapy or disease modeling.^16^, ^17^ Human iPSCs also hold promise for enhancing regenerative strategies in CHD by enabling the creation of engineered heart tissues and leveraging bioengineering methodologies, including the fabrication of 3D cell patches.^18^ Furthermore, human iPSCs provide an innovative platform for modeling CHD, allowing for the study of etiologies and the development of complications through the de novo generation of patient‐specific cardiomyocytes, endocardium, or cardiac organoids.^19^, ^20^, ^21^ The utilization of stem cells in CHD treatment, along with the associated clinical challenges and future applications, has been extensively explored in other reviews and meta‐analyses.^22^, ^23^, ^24^, ^25^

Besides stem cells, alternative cardiac regenerative strategies garner growing research interest, aiming toward the ultimate goal of fully repairing heart function.^26^ One promising avenue of research involves investigating the stimulation of cardiomyocyte proliferation as a potential therapeutic approach,^27^, ^28^ yet its application in CHD treatment remains largely unexplored. The regenerative capacity of proliferative cardiomyocytes is particularly prominent during the neonatal period but gradually diminishes with age, while ongoing research continues to elucidate the precise mechanisms driving this phenomenon.^29^, ^30^, ^31^, ^32^ Increasing evidence has begun to unveil the pivotal role of cardiomyocyte proliferation in heart development and its relevance to CHD.^33^, ^34^, ^35^ The intricate relationship between cardiomyocyte proliferation and maturation is crucial in understanding the developmental pathogenesis and postnatal progression of CHD, highlighting its pivotal role in elucidating molecular mechanisms underlying these congenital cardiac anomalies and guiding the development of innovative therapeutic interventions. In this review, we explore the latest advancements in understanding human cardiomyocyte proliferation and its implications for the pathogenesis and progression of CHD. Furthermore, we highlight promising approaches aimed at boosting cardiomyocyte proliferation or regeneration for potential therapeutic interventions in CHD.

## 2 CHARACTERISTICS OF PROLIFERATIVE CARDIOMYOCYTES DURING DEVELOPMENT AND IN NEWBORNS

Understanding the pivotal signaling pathways involved in human cardiomyocyte proliferation holds promise for advancing our understanding of heart development and potentially uncovering novel therapeutic targets for the treatment of CHD. It is widely recognized that de novo cardiomyocytes originate from the differentiation of early cardiac progenitors and undergo proliferation during mammalian human development.^36^ Robust cardiomyocyte proliferation is essential for the proper formation of cardiac structures, including myocardial trabeculation and ventricular walls. The proliferation process necessitates tight regulation of extracellular growth factor levels, as well as intracellular expression of transcription factors to modulate cell cycle regulators. The key pathways underlying embryonic cardiomyocyte proliferation include neuregulin, bone morphogenetic protein, neurogenic locus Notch homolog protein (NOTCH), Hippo/Yap, IGF/PI3K, and Wnt, as summarized in other reviews.^35^, ^36^, ^37^ Notably, some of these factors or their inhibitors have been utilized in diverse combinations to mimic heart development environments, prompting the differentiation of human iPSCs into cardiomyocyte lineages or cardiac organoids.^38^ These models would offer authentic platforms for investigating the intricate interplay between human cardiomyocyte proliferation and CHD through aberrant signaling regulation or gene mutations.

During development, cardiomyocyte proliferation contributes significantly to heart growth and structural formation, particularly after the heart tube loops.^39^ Proliferation and cell division of cardiomyocytes ensures proper formation of heart chambers including atrial and ventricular walls. Once the formation of the four chambers is visible, cardiomyocyte proliferation rates vary locally within the developing heart. Proliferation slows down in the outflow tract (the region where blood exits the heart), while the inflow tract and atria (upper chambers) continue to show high rates of cell division.^40^ The complex interplay between crucial molecular signals (such as BMP, WNT, NOTCH, and Hippo/YAP) and cardiogenic transcription factors (such as NKX2‐5, GATA4, MEF2C, and TBX5) is essential for coordinating cardiomyocyte proliferation with the structural formation of the heart.^36^ The formed embryonic myocardial walls, including the endocardium and epicardium, provide critical growth factor cues and structural supports for directing progenitor epithelial‐to‐mesenchymal transformation, migrations, and differentiations into fibroblasts, coronary vascular smooth muscle cells, and/or endothelial cells.^41^, ^42^ This process leads to the formation of a functional cardiac septum, valves, and large blood vessels.^43^ Thus, cardiomyocyte proliferation and cell division play an essential role in heart morphological formation and function during development under physiological conditions.

Embryonic or fetal cardiomyocytes, once differentiated, persist in proliferating within the functioning organ throughout prenatal development. After birth, neonatal proliferative cardiomyocytes transition primarily to hypertrophic growth and undergo maturation in response to cues or factors such as increased blood flow, aerobic environments, and metabolic changes.^44^, ^45^, ^46^ This shift is characterized by changes in contractile protein isoforms from fetal to adult, metabolic adaptation from glycolytic to oxidative, repression of cell cycle activators, and upregulation of cell cycle inhibitors.^46^, ^47^ These genetic, morphological, and functional traits associated with cell cycling activation and cell division can also serve as key indicators guiding the technical refinement of methods for detecting cardiomyocyte proliferation.^27^, ^48^, ^49^ Research on neonatal mammals, including mice, pigs, and large animals, indicates that the proliferative window of cardiomyocytes extends from 1 day to 1 week after birth, after which the rate of cardiomyocyte proliferation (division or cytokinesis) will decline to extremely low level or be barely detectable.^30^, ^49^, ^50^, ^51^, ^52^, ^53^, ^54^

Emerging evidence also reveals that while human infants and children exhibit notable cardiomyocyte proliferation, this activity diminishes significantly or becomes undetectable in adult humans.^55^, ^56^, ^57^ Interestingly, the study from Dr. Kühn's lab demonstrated that humans have the capacity to generate new cardiomyocytes from birth until around 20 years of age, with a notable 3.4‐fold increase, until the heart reaches its adult size.^56^ However, research from Dr. Frisén's lab has revealed that the number of cardiomyocytes remains relatively stable in growing hearts or with age, as evidenced by samples obtained from donors ranging from 1 month to 73 years old, with a turnover rate of less than 1%.^57^ This inconsistency in results may arise from the utilization of varying measurement techniques, but they consistently demonstrate the trend of cardiomyocyte proliferation rate peaking in early childhood and gradually declining in adulthood. Indeed, a case report highlighted the remarkable ability of a newborn child with severe myocardial infarction (caused by coronary artery occlusion detected within the first day after birth) to repair myocardial damage and fully restore cardiac function, followed by critical care interventions.^58^ While the precise mechanism remains unclear, it is speculated that this phenomenon may involve cardiomyocyte proliferation, as observed in rodent studies.

## 3 ROLES OF CARDIOMYOCYTE PROLIFERATION IN CHD PATHOGENESIS DURING DEVELOPMENT

Considering the processes of abnormal heart development in CHD, it is conceivable that abnormalities in cardiomyocyte proliferation or cell cycling could lead to the formation of septal defects, valve anomalies, and structural alterations in cardiac tissue.^33^, ^34^ Human iPSC‐derived cardiomyocytes or organoids offer potential as disease modeling tools without the ethical concerns associated with human embryo use. The cardiomyocytes differentiated from iPSCs derived from samples of patients with pulmonary atresia exhibited reduced proliferative capacity or cell cycle progression, but they demonstrated a more advanced metabolic profile in mitochondrial respiration, suggesting a potential developmental mechanism underlying CHD‐related metabolic dysfunction.^59^ Additionally, iPSC‐cardiomyocytes derived from individuals with Down syndrome displayed reduced proliferation and migration capacity in response to the elevated expression of type VI collagen.^60^ However, the molecular mechanism underlying cardiomyocyte proliferation's contribution to CHD remains largely elusive, primarily due to the pathological heterogeneity and complex etiologies involved. Further validation of the role of cardiomyocyte proliferation in CHD development is imperative, requiring direct clinical evidence and human specimens.

### 3.1 Genetic mutations related to defective cardiomyocyte proliferation

**FIGURE 2:**
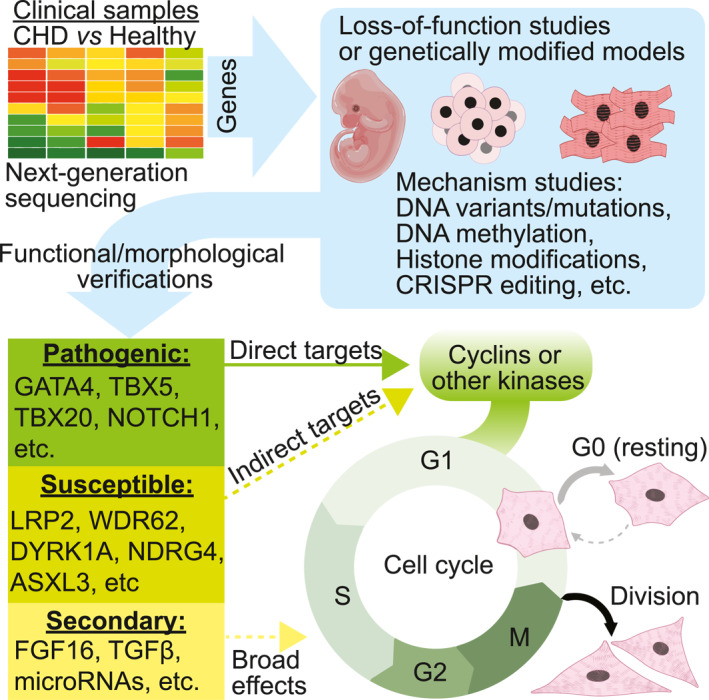
Genetic or epigenetic alterations related to CHD during heart development. Genetic and epigenetic alterations underlying CHD can be identified using next‐generation sequencing techniques. Functional validation through loss‐of‐function studies or genetic manipulation models can classify the involved genes into (1) pathogenic genes (These typically encode cardiogenic transcription factors that directly regulate cardiomyocyte cell cycle activity in CHD through well‐established mechanisms), (2) susceptibility genes (such as pathway receptors or kinases, indirectly influence cardiac cell cycling and contribute to an increased risk of CHD), and (3) secondary genes (such as growth factors or microRNAs, have broader effects on cell cycling and may not be specific to the heart but can influence overall cell proliferation). CHD, congenital heart disease.

Genetic abnormalities have emerged as the predominant factors driving the pathogenesis of CHD, a notion demonstrated by substantial progress in genome sequencing and the accessibility of comprehensive datasets pertaining to the human CHD genome.^61^, ^62^, ^63^ Other excellent reviews have thoroughly discussed the roles of gene mutations and variants, both coding and noncoding, in abnormal heart morphogenesis that contribute to the pathogenesis of CHD.^64^, ^65^, ^66^ However, pinpointing specific defects remains challenging due to the intricate genetic nature of CHD. Considering the crucial role of cardiomyocyte proliferation in heart development, mutations in associated genes could give rise to morphological abnormalities and contribute to the pathogenesis of CHD. Specifically, gene mutations associated with reduced cardiomyocyte proliferation may play a role in myocardial hypoplasia observed in CHD. Therefore, our discussion focuses on human genetic mutations that impact pathways governing cardiomyocyte cell cycle and proliferation in this section (Figure 2).

Intracellular pathways mediated by receptor kinases play a critical role in orchestrating cardiomyocyte differentiation and proliferation throughout heart development. Predicted damaging variants in the *LRP2* (LDL receptor‐related protein) gene were found to be more prevalent in a cohort of individuals with hypoplastic left heart syndrome (HLHS) or TOF and associated with impaired cardiomyocyte proliferation, underscoring the need for further investigation to elucidate their specific role in the pathogenesis of CHD.^67^, ^68^ Through sequencing analysis in a large cohort of individuals with CHD, *WDR62* (WD repeat‐containing protein 62) emerged as a susceptibility gene harboring high‐frequency variants associated with CHD.^69^ Further investigation revealed that *WDR62* played a crucial role in cardiomyocyte proliferation by interacting with the key mitotic kinase aurora kinase A, which mediates spindle assembly. Human fetal hearts affected by Down syndrome exhibited decreased expression of mitochondrial respiration and cell proliferation genes, correlating with increased copies of the dual‐specificity tyrosine phosphorylation‐regulated kinase 1A (*DYRK1a*) gene.^70^ A compound heterozygous mutation in the *ASXL3* (ASXL Transcriptional Regulator 3) gene identified from CHD samples was discovered to promote cell apoptosis or suppress cell proliferation in human cardiomyocytes by inhibiting the Ras/extracellular signal‐regulated kinase signaling pathway.^71^ A pathogenic variant of the N‐myc downstream‐regulated gene4 has been identified in patients with VSD or TOF, and it is associated with impaired human cardiomyocyte proliferation and cell‐cycle arrest.^72^

A network of cardiac transcription factors critically regulates cardiomyocyte proliferation by controlling the expression of downstream genes involved in cell cycling. *NOTCH1* is a critical gene for promoting cardiomyocyte cycling or proliferation and its pathogenic mutations or deficiencies are associated with severe forms of CHD such as HLHS and TOF.^73^, ^74^, ^75^ Knockout of *NOTCH1* gene may result in skewed differentiation of human cardiac progenitors, inhibition of ventricular development, and impaired proliferation, collectively contributing to left ventricular hypoplasia in CHD.^76^ In addition, the *GATA4* (GATA binding protein 4) gene mutations that are responsible for ASD can lead to impaired proliferation of human cardiomyocytes through downregulation of a growth factor FGF16.^77^ Depletion of genes such as *GATA4* and *TBX5* can disrupt cyclin‐dependent kinases, leading to defects in cardiomyocyte proliferation and atrioventricular septation.^78^ Nonsense and missense germline mutations within the T‐box DNA‐binding domain of human *TBX20* are associated with a family history of CHD and a diverse array of developmental anomalies, with loss‐of‐function studies of *TBX20* indicating reduced cardiomyocyte proliferation and cell cycle arrest during heart development.^79^, ^80^ Interestingly, genome correction of a *TBX20* mutation and inhibition of its downstream TGF‐beta signaling were adequate to reverse the disease phenotype of left ventricular non‐compaction.^81^

While recent advancements in next‐generation genome sequencing have unveiled novel genetic etiologies of CHD, elucidating their causative contributions and validating their mechanisms in cellular and animal model systems remains a significant challenge. The emergence of clustered regularly interspaced short palindromic repeats (CRISPR) technology, which enables precise genomic DNA cleavage guided by a small set of guide RNAs, presents a potent tool for genome editing.^82^ CRISPR technology has been employed to model, create, or correct complex inherited genetic mutations, such as base substitutions, deletions, and insertions, commonly associated with CHD.^83^, ^84^, ^85^ Although CHD‐related cell lines or animal models have been generated or modified with mutations in several cardiac genes using CRISPR technology,^86^, ^87^, ^88^ the influence of these genetic modifications on cardiomyocyte proliferation remains underexplored. It is anticipated that CRISPR tools will be increasingly utilized to investigate the influence of inherited and de novo mutations, as well as noncoding genetic variants, on cardiomyocyte differentiation and proliferation in various CHD subtypes. This trend is driven by the advantages of CRISPR technology, such as precise targeting, high efficiency, scalability for multiplexed targets, and ease of manipulation, particularly for human genes crucial in cardiogenesis.^89^

### 3.2 Epigenetic mechanisms associated with impaired cardiomyocyte growth

A mutation in a single cardiac gene or transcription factor involved in cardiogenesis is adequate to account for approximately 10% of all CHD cases, implying that extragenomic factors may exert a predominant influence on CHD pathogenesis.^90^ Epigenetic modifications are conservative extragenomic mechanisms that do not involve alterations in the DNA sequence but possess the ability to regulate gene expression by influencing the transcription machinery within cells.^91^ Significantly, whole‐genome bisulfite sequencing of cord blood from monozygotic twins, who are essentially genetically identical but discordant for CHD, revealed a panel of differentially methylated genes associated with CHD.^92^, ^93^ Emerging evidence underscores the correlations or interactions between environmental risk factors, genetic variants (mutations), and epigenetic mechanisms, contributing to abnormal cardiac differentiation or the development and onset of CHD.^94^, ^95^, ^96^, ^97^ In this review, our primary focus lies on exploring aberrant epigenetic mechanisms linked to impaired human cardiomyocyte proliferation, a factor contributing specifically to the pathogenesis of CHD in preclinical and clinical contexts (Figure 2). The discovery of novel epigenetic modifiers in human cells holds significant promise for the development of innovative strategies in diagnosing or treating CHD.

Among epigenetic modifications, extensive investigation has focused on DNA methylation in a large cohort of patients with CHD, underscoring its substantial potential in personalized medicine. This includes its utility in prediagnosis and prognosis assessment of CHD through profiling various specimen sources such as blood samples, placental tissues, buccal swabs, and cfDNAs.^98^, ^99^, ^100^, ^101^ Mechanistically, promoter region hypermethylation can downregulate the expression of genes crucial for normal cardiac development, thereby contributing to the pathogenesis of CHD. For example, elevated methylation levels at the NOTCH4 or VANGL2 promoters have been linked to reduced expressions in patients with TOF, potentially due to interference with the gene transcription machinery.^102^, ^103^ Furthermore, hypermethylation of other key genes, such as *MSX1* and *GATA4*, has been observed in the DNA of developing hearts in fetuses with both isolated and syndromic heart malformations.^104^ Pyrosequencing of peripheral blood samples revealed that methylation of the *TBX20* gene could serve as a potential risk marker for congenital septal defects and PDA, with additional environmental risk factors including vitamin consumption and maternal infections being implicated.^105^ Apart from cardiogenic genes or transcription factors, DNA methylation abnormalities of imprinted genes are also linked to CHD.^106^, ^107^ In addition to aberrant methylation of promoter CpG islands affecting gene expression levels, methylation changes may also induce novel, differential splicing events among sarcomeric genes and alter transcription factor binding sites in patients with CHD.^108^ Additional epigenetic mechanisms, such as histone posttranslational modifications, have been observed to vary depending on the genetic mutation of related modifier genes, as evidenced by high‐throughput sequencing studies on human CHD samples.^109^, ^110^, ^111^ However, further investigation is needed to fully elucidate their specific contributions to CHD pathogenesis. Multimodal epigenetic mechanisms have been implicated in the processes of cardiomyocyte proliferation or cell cycle reentry,^112^, ^113^ but it remains unclear whether these integrated mechanisms are also involved in impaired cardiomyocyte growth in CHD, warranting further study in this context.

Additionally, noncoding RNAs play crucial roles in epigenetic mechanisms by serving as guides, scaffolds, or decoys that influence gene expression patterns, thus leading to cellular phenotype changes associated with heart diseases.^114^, ^115^ Recent studies indicate the participation of noncoding RNAs, including microRNAs and long noncoding RNAs (lncRNAs), in the regulation of cardiomyocyte proliferation, thereby contributing to the pathogenesis of CHD. Deep sequencing of human heart tissues from prevalent CHD subtypes, such as ASD, VSD, and TOF, unveiled a miRNome panel comprising 295 dysregulated miRNAs, several of which are linked to cardiogenesis, cardiomyocyte proliferation, or cardiac dysfunction.^116^ Furthermore, miR‐29, which was significantly elevated in patients with right ventricular outflow tract defects, was found to modulate cardiomyocyte proliferation by targeting NOTCH2.^117^ An lncRNA uc.457, found to be differentially expressed in cardiac tissue from patients with VSD, exhibited inhibitory effects on cardiomyocyte proliferation by suppressing maturation‐associated genes including histone cell cycle regulator, natriuretic peptide A, cTnT, and Mef2c.^118^ A study on ASD revealed that miR‐19 was mediated by the NKX2‐5 mutation that contributed to aberrant cardiomyocyte proliferation.^119^ Importantly, the identification of RNA biomarkers from fetal extracellular vesicles isolated from maternal blood emerges as a novel strategy for pre‐diagnosis or outcome prediction of CHD.^120^, ^121^, ^122^ Although numerous noncoding RNAs have been identified in association with CHD from patient specimens, further investigation is necessary to clarify their precise role in cardiomyocyte proliferation or CHD pathogenesis and to determine whether a causative relationship exists.

Significantly, the engineering of CRISPR‐associated protein 9 (Cas9) into a deactivated form known as dead Cas9 (dCas9), which lacks endonuclease activity but retains its ability to bind to target sequences without cleaving DNA, along with the CRISPR/dCas9 system, offers a programmable platform for precise gene regulation (activation or repression) and epigenome controls.^123^, ^124^, ^125^ Recently, the CRISPR/dCas9 technology has been leveraged to modulate epigenetic mechanisms, including DNA methylation, histone modifications, and noncoding RNAs.^126^, ^127^, ^128^ However, these innovative tools for epigenetic editing have yet to be widely employed in CHD research. Given the incomplete understanding of epigenetic mechanisms underlying the proliferation of CHD‐derived cardiomyocytes, utilizing CRISPR/dCas9‐based epigenetic editing tools presents unprecedented opportunities to investigate these aspects. Further exploration in cell or animal models will shed light on the role of epigenetic mechanisms in CHD pathogenesis, particularly in processes related to cardiomyocyte differentiation and proliferation, and pave the way for identifying new epigenetic targets in the quest for novel therapeutic strategies.

## 4 POSTNATAL CARDIAC MULTINUCLEATION AND POLYPLOIDIZATION IN CHD

After birth, proliferating cardiomyocytes undergo hypertrophic growth and functional maturation characterized by cell cycle exit, multinucleation, or polyploidization in healthy infants.^129^ While numerous studies have highlighted the role of defective cardiomyocyte proliferation in the onset of CHD during embryogenesis, there remains a notable gap in the investigation of the dynamic process of cardiomyocyte cell cycle activity and cytokinesis in infants with CHD after birth. Interestingly, cardiomyocytes from infants with CHD under 3 months old exhibited heightened proliferative potential compared to older infants with CHD, yet demonstrated lower cell cycle activity compared to controls without heart disease.^130^, ^131^ These studies suggested that not all cardiomyocytes undergo terminal differentiation or permanent cell cycle withdrawal shortly after birth, or that at least a subset retains cycling capacities during early infancy. However, active cell cycle activity or high DNA contents in these CHD conditions do not inevitably lead to progression to karyokinesis and/or cytokinesis, potentially resulting in polyploidy (*via* endocycling) and/or multinucleation (*via* endomitosis).^132^

**FIGURE 3:**
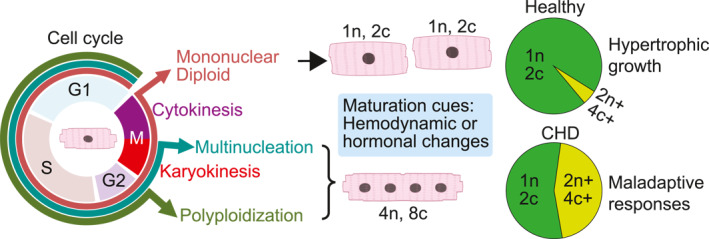
Defective cardiomyocyte proliferation and maturation in CHD after birth. In contrast to healthy subjects, CHD infants exhibit a reduced number of mononuclear, diploid cardiomyocytes, but increased cell populations with multinucleation and polyploidization due to impaired cell cycle regulation during development. After birth, these abnormal cardiomyocytes in CHD patients respond poorly to cues that trigger healthy heart growth and maturation (developmental hormones or increased workload). In contrast, healthy cardiomyocytes undergo a controlled enlargement (hypertrophic growth) and improve their function as the body grows. CHD, congenital heart disease.

Cardiomyocytes in infants with HLHS exhibited a significant rise in polyploid nuclei at the expense of diploid nuclei compared to healthy infants, indicating premature cell cycle arrest in CHD during the differentiation of early cardiac progenitor lineages.^133^ In contrast to healthy infants, who exhibit an adequate number of cardiomyocytes in response to postnatal growth cues, individuals with CHD characterized by cardiomyocyte hypoplasia experience maladaptive responses to these cues, rendering them vulnerable to adverse conditions (Figure 3). For example, hemodynamic overload and hypoxemia during TOF may accelerate ontogenetic growth and induce immature differentiation of cardiomyocytes after birth, characterized by polyploidy, increased cell size, and disordered gap junctions, without reactivating proliferation.^134^ Besides hemodynamic cues, the single‐cell and 3D modeling study utilizing iPSC‐cardiomyocytes with an HLHS genetic background revealed that premature alterations in ventricular hypoplasia hindered normal tissue responses to developmental signals for postnatal growth, resulting in the accumulation of DNA damage and increased apoptosis.^133^ Analysis of a cohort of TOF heart samples revealed that the generation of bi‐ or multi‐nucleated cardiomyocytes can be generated within the first 6 months after birth and then persisted for at least 10 years, which is a phenotype rarely observed in healthy humans, indicating the occurrence of multiple serial cytokinesis failures in CHD.^135^ The molecular mechanism underlying cardiomyocyte cytokinesis failure in TOF could be linked to reduced expression of *ECT2* gene, a crucial regulator of cytokinesis that initiates cleavage furrow constriction.^135^, ^136^

It is important to note that variations in postnatal cardiomyocyte nucleation or polyploidy may be contingent upon the etiology of the different types of CHD, and whether this mechanism can be universally applied remains inconclusive. Additionally, it is uncertain whether the observed mechanism or gene expression changes in infants with particular CHD subtypes result from differences in developmental stages or are consequences of heart disease, given the limited access to heart tissue from healthy infants and children for comparison as age‐matched controls. Encouragingly, advancements in bioengineered tissue techniques offer pro‐maturational cues capable of mimicking postnatal signals, facilitating the identification of abnormal developmental trajectories and functional defects in CHD‐derived iPSC‐cardiomyocytes.^137^ Furthermore, the presence of public biobanks offers promise by facilitating the generation of human iPSC‐derived cardiomyocytes from diverse genetic backgrounds, including both CHD and non‐disease cohorts, enabling comparisons at equivalent developmental stages using standardized differentiation or 3D engineering protocols. Such endeavors hold the potential for verifying the mechanisms underlying cardiomyocyte cycling, nucleation, or polyploidy across a broader spectrum of CHD genetic backgrounds.

## 5 APPROACHES TO ENHANCE CARDIOMYOCYTE PROLIFERATION FOR CHD REPAIR

The corrective surgery for CHD in infancy carries inherent risks associated with the procedure or device, often resulting in complications such as embolic stroke, hypoxic‐ischemic injuries, and subsequent developmental and cognitive delays, including brain injuries.^138^, ^139^, ^140^, ^141^ Therefore, implementing a strategy to alleviate clinical manifestations, postpone surgery, or prevent complications may be preferable and allow the infant to grow and better tolerate the surgical procedure and subsequent healing process. Regenerative medicine, particularly the stimulation of cardiomyocyte proliferation, is emerging as an appealing approach for generating new myocardium to repair defects associated with CHD. The potential targets for stimulating cardiomyocyte proliferation in CHD therapies are outlined in Table 1.

**TABLE 1:** Pharmacological targets for enhancing cardiomyocyte proliferation in CHD.

Therapeutics	Mechanisms	CHD models	Outcomes	Reference
Propranolol	Blocking β‐adrenergic receptors‐Hippo signaling, and restoring ECT2 expression	Cardiomyocytes from TOF infants	Enhancing cytokinesis	135
Recombinant growth factor neuregulin‐1 (rNRG1)	Activating tyrosine kinase receptor, ErbB4	Cardiomyocytes from TOF infants	Stimulating M‐phase entry	131
Leucettinib‐21	Inhibition of *DYRK1A* kinase activity	Down syndrome mouse embryos	Reversing expressions of proliferative genes	70
SD208, RepSox	Inhibition of TGFβ signaling	Patient‐derived iPSCs of left ventricular non‐compaction cardiomyopathy	Enhancing cell cycling	81

Abbreviations: CHD, Congenital heart disease; rNRG1, recombinant growth factor neuregulin‐1; TOF, tetralogy of fallot.

Dr. Kühn's lab demonstrated *ECT2* gene as a novel target for alleviating the mitotic block and promoting the division of bi‐ or multi‐nucleated cardiomyocytes into new mononuclear myocytes to repair defects associated with TOF.^135^ Their mechanistic study further indicated that *ECT2* gene expression was regulated by the YAP1‐TEAD1/2 transcription factor complex, thereby reinforcing the pivotal role of the Hippo‐Yap signaling pathway in cardiomyocyte proliferation, which is consistent with findings from previous studies.^142^, ^143^ Indeed, YAP1 mRNA and protein levels, as well as nuclear localization, exhibited a significant decrease in VSD specimens (from donors approximately 6 months old) when compared to normal heart samples within the same age range.^144^ Therefore, identifying the upstream receptors that regulate the Hippo‐Yap pathway is essential for the development of pharmacological interventions aimed at modulating cardiomyocyte proliferation. Significantly, Dr. Kühn's lab discovered that treatment with propranolol (a β‐adrenergic receptor blocker)‐augmented cardiomyocyte cytokinesis or division, as indicated by the heightened presence of ECT2‐positive midbodies and reduced binucleation in cardiomyocytes isolated from patients with TOF.^135^ This discovery has motivated them to launch a clinical trial (clinicaltrials.gov NCT04713657) assessing the therapeutic impact of administering propranolol on cardiomyocyte division and generation in infants with TOF, with anticipated results expected in 2026.^145^ Of note, β blockers, such as carvedilol and propranolol, have not demonstrated significant improvements in heart failure outcomes in children and adolescents with CHD compared to placebo in clinical trials, although earlier initiation of the medical therapy may confer benefits in infants with CHD.^146^, ^147^, ^148^ Thus, the advancement of pharmacological therapeutics targeting cardiomyocyte cell cycling pathways has the potential to bring in a novel paradigm of integrated medical and surgical interventions for infants with CHD.

It is also noteworthy that genetic depletion of β‐adrenergic receptors or treatment with propranolol did not alter M‐phase activity in cardiomyocytes,^135^ indicating that β‐blockers promoted cardiomyocyte proliferation by mitigating cytokinesis failure rather than inducing de novo cycling activity. Given the significantly low cell cycle activity of CHD cardiomyocytes, increasing cell cycling activity before the M‐phase would further increase the number of cardiomyocytes. However, this integrated strategy has not been investigated in current studies of CHD. Furthermore, postnatal cardiomyocyte proliferation is intricately regulated by the contextual interplay between the downregulation of essential cell cycle factors and the upregulation of cell cycle inhibitors, orchestrated by complex signaling networks including Hippo, Wnt/β‐catenin, Notch, and PI3K/protein kinase B pathways.^29^, ^131^ Therefore, a single regulator may not suffice to govern multiple pathways or overcome the diverse barriers hindering cardiomyocyte cell division. Despite the identification of various genes, proteins, or noncoding RNAs with the potential to stimulate cardiomyocyte cycling and proliferation, challenges persist in gene delivery approaches, hindering their clinical application.^29^, ^149^ High‐throughput screenings of synthetic molecule libraries, particularly FDA‐approved drugs, have been conducted in human iPSC‐derived cardiomyocytes to evaluate drug‐induced cardiotoxicity.^150^ Recently, FDA‐approved drugs have undergone screening in neonatal rat ventricular myocytes to identify effective molecules with the potential to stimulate cardiomyocyte proliferation and facilitate regeneration.^151^ Hence, these broad screening methods offer potential in identifying novel compounds capable of robustly inducing cardiomyocyte proliferation, including completing cell cycling and cytokinesis, within the diverse contexts of human iPSC‐cardiomyocytes derived from various CHD subtypes. Further in vitro and in vivo investigations are warranted to validate these findings.

## 6 ADVANTAGES AND DISADVANTAGES OF CARDIOMYOCYTE PROLIFERATION THERAPY

The emerging pharmacological approaches to stimulate the proliferation of preexisting cardiomyocytes offer promising adjunct therapeutics for treating CHD, complementing traditional treatments such as surgical interventions. The combined paradigm of medical and surgical approaches has the potential to revolutionize the management of patients with CHD, currently being assessed in the ongoing clinical trial.^145^ Further clinical investigations will also provide opportunities to evaluate the impact of cardiomyocyte proliferation therapy on major adverse cardiovascular events often associated with traditional surgical interventions, such as arrhythmia, pulmonary hypertension, and heart failure in CHD. Insights from other heart diseases may offer valuable extrapolations regarding the potential benefits of cardiomyocyte proliferation in mitigating these adverse events in CHD. Excessive cardiac fibrosis or scarring, resulting from numerous cardiomyocyte losses in CHD or ischemic heart disease, can frequently lead to arrhythmias and heart failure.^152^, ^153^ Therefore, fibrosis could be directly mitigated by replacing scar tissue with an increased number of functional cardiomyocytes through cell cycle reentry and stimulated proliferation.^154^ This mechanism of action may have broad applicability in CHD treatment. Cardiac output or pump function could also be improved by enhancing the cell cycle activity of functional cardiomyocytes,^155^ though it remains to be determined whether such improvement can potentially alleviate the pressures in the pulmonary arteries associated with pulmonary hypertension in CHD.

Despite promising approaches, cardiomyocyte proliferation therapy faces limitations related to efficiency and specificity (safety) that require further investigation in regenerative medicine. Current proliferation stimulation approaches remain insufficient to fully regenerate the diseased heart.^156^ Additionally, newly regenerated cardiomyocytes could be heterogeneous and possess immature structural and functional properties, potentially hindering integration with existing tissue. It is crucial to control cell proliferation to prevent unwanted consequences such as increased heart mass or arrhythmias.^157^ Encouragingly, transient and cardiomyocyte‐specific delivery of cell cycle inducers is a promising tool to safely induce cardiomyocyte proliferation without the development of cardiac arrhythmias or systemic tumorigenesis.^158^ Therefore, developing cardiac subtype‐specific targeted vectors could address the safety limitations of cardiomyocyte proliferation therapy. Furthermore, single‐nucleus RNA sequencing offers a comprehensive view of cardiac cell phenotypes, paving the way for personalized treatments for CHD.^159^ Thus, rigorous clinical trials and continued research are necessary to fully elucidate the efficacy and safety of targeted cardiomyocyte proliferation therapy.

## 7 PERSPECTIVES AND CONCLUSION

Precise regulation of cardiomyocyte proliferation and maturation is crucial for the development of a four‐chambered heart. During embryogenesis, the heart primarily undergoes growth through cardiomyocyte hyperplasia, a process susceptible to influence by genetic variants, epigenetic alterations, and/or environmental factors (alcohol, drugs, infections/immunities, nutrition, etc.), ultimately contributing to CHD pathogenesis (Figure 1). In addition to animal models, the technological advancements in generating human iPSC‐derived cardiomyocytes and cardiac organoids offer a crucial platform for investigating the etiology of CHD. Moreover, the development of CRISPR‐based genome or epigenome editing technologies opens up new avenues to explore the mechanisms underlying defective cardiac differentiation and proliferation in CHD, as well as to assess the diagnostic value of new biomarkers (genetic variants, proteins, noncoding RNA, etc.). These advancements can be translated into clinical practice through the approaches of in vitro diagnostics on tissue or blood samples (DNA, RNA, cfDNA, microRNAs, extracellular vesicles, etc.), thereby providing additional benefits for accurate fetal cardiac diagnosis or prenatal screening of CHD, in combination with prenatal ultrasound or MRI. These efforts advance prenatal CHD diagnosis, allowing for early counseling, informed decision‐making, and preparation for postnatal management or termination of pregnancy.

Postnatal cardiomyocyte proliferation has been observed in infants with CHD, albeit at lower rates compared to healthy infants, and is notably absent in adults. Expanding the clinical dataset by gathering additional cases of human infants or children will aid in mapping the precise time window during which cardiomyocytes retain proliferative capacities critical for optimal cardiac healing. Several novel approaches have been developed for isolating primary human cardiomyocytes or imaging cycling cells in CHD heart tissues,^48^, ^160^ which will facilitate the study of cardiomyocyte proliferative capacity in infant samples obtained from surgeries. Advances in next‐generation sequencing and single‐cell analysis promise to offer deeper insights into the mechanisms or processes underlying human cardiomyocyte proliferation and maturation postnatally. Moreover, integrating CHD‐derived cardiomyocytes with tissue engineering approaches will yield a high‐fidelity model to mimic developmental and maturation cues, facilitating the investigation of cardiomyocyte proliferation or cell cycle regulation. Pharmacological therapeutics targeting cardiomyocyte cell cycle pathways show promise for translation into clinical use in CHD therapy, circumventing safety concerns associated with gene delivery. Controlled medical administration can also mitigate unrestrained cardiac growth in the long term. Furthermore, iPSC‐derived cardiomyocyte models offer a crucial platform for screening effective cell cycle regulators from drug libraries and evaluating their potential impact on multinucleation and polyploidization across various CHD subtypes. Pharmacological therapies, administered as early as possible before surgical interventions and continued afterward, may provide improved healing outcomes for CHD infants, necessitating long‐term follow‐up studies to evaluate prognosis.

In summary, we delve into the latest advancements in genetic or epigenetic mechanisms related to defective cardiomyocyte proliferation in CHD throughout development and after birth. Targeting cardiomyocyte cell cycle pathways shows promise in enhancing cardiomyocyte cytokinesis, division, and regeneration for repairing heart defects. Progress in human iPSC techniques, including heart tissue engineering and cardiac organoids, along with CRISPR‐based genome and epigenome editing, and single‐cell sequencing technologies, will accelerate the exploration of abnormal machinery underlying cardiomyocyte differentiation, proliferation, and maturation across various genetic backgrounds of CHD. Ongoing studies on screening drugs that regulate cardiomyocyte cell cycling are poised to translate this nascent technology of enhancing cardiomyocyte proliferation into a new therapeutic paradigm for CHD surgical interventions.

## AUTHOR CONTRIBUTIONS

**Jialiang Liang**: The manuscript writing and the final approval of the manuscript. **Xingyu He**: The manuscript editing. **Yigang Wang**: The manuscript editing and the final approval of the manuscript.

## CONFLICT OF INTEREST STATEMENT

The authors indicated no potential conflicts of interest.

## ETHICS STATEMENT

No ethical approval was required as this is a review article with no original research data.

## Data Availability

Data sharing not applicable to this article as no datasets were generated or analyzed during the current study.
